# Lightweight and Low-Parametric Network for Hardware Inference of Obstructive Sleep Apnea

**DOI:** 10.3390/diagnostics14222505

**Published:** 2024-11-08

**Authors:** Tanmoy Paul, Omiya Hassan, Christina S. McCrae, Syed Kamrul Islam, Abu Saleh Mohammad Mosa

**Affiliations:** 1 Department of Electrical Engineering and Computer Science, University of Missouri, Columbia, MO 65211, USA; tanmoy.paul@health.missouri.edu (T.P.); omiyahassan@boisestate.edu (O.H.); islams@missouri.edu (S.K.I.); 2 Department of Biomedical Informatics, Biostatistics, and Medical Epidemiology, School of Medicine, University of Missouri, Columbia, MO 65211, USA; 3 School of Nursing, University of South Florida, Tampa, FL 33620, USA; christinamccrae@usf.edu

**Keywords:** apnea, depth-wise separable convolution, transfer learning, model fusion, energy efficient AI

## Abstract

**Background**: Obstructive sleep apnea is a sleep disorder that is linked to many health complications and can even be lethal in its severe form. Overnight polysomnography is the gold standard for diagnosing apnea, which is expensive, time-consuming, and requires manual analysis by a sleep expert. Artificial intelligence (AI)-embedded wearable device as a portable and less intrusive monitoring system is a highly desired alternative to polysomnography. However, AI models often require substantial storage capacity and computational power for edge inference which makes it a challenging task to implement the models in hardware with memory and power constraints. **Methods**: This study demonstrates the implementation of depth-wise separable convolution (DSC) as a resource-efficient alternative to spatial convolution (SC) for real-time detection of apneic activity. Single lead electrocardiogram (ECG) and oxygen saturation (SpO_2_) signals were acquired from the PhysioNet databank. Using each type of convolution, three different models were developed using ECG, SpO_2_, and model fusion. For both types of convolutions, the fusion models outperformed the models built on individual signals across all the performance metrics. **Results**: Although the SC-based fusion model performed the best, the DSC-based fusion model was 9.4, 1.85, and 11.3 times more energy efficient than SC-based ECG, SpO_2_, and fusion models, respectively. Furthermore, the accuracy, precision, and specificity yielded by the DSC-based fusion model were comparable to those of the SC-based individual models (~95%, ~94%, and ~94%, respectively). **Conclusions**: DSC is commonly used in mobile vision tasks, but its potential in clinical applications for 1-D signals remains unexplored. While SC-based models outperform DSC in accuracy, the DSC-based model offers a more energy-efficient solution with acceptable performance, making it suitable for AI-embedded apnea detection systems.

## 1. Introduction

Obstructive sleep apnea (OSA) is a prevalent sleep-related breathing disorder caused by the collapse of the upper airway, resulting in disrupted airflow. This repetitive blockage of the upper airway causes breathing interruptions called hypopnea and apnea, characterized by reduced airflow and complete cessation of breathing for at least 10 s, respectively. Hypopnea is also accompanied by a decrease in blood oxygen levels by at least 4% [1,2,3]. Individuals with moderate to severe apnea may experience numerous such apneic events during the night, leading to detrimental health effects. Daytime fatigue caused by frequent awakenings is the most common effect of OSA [4]. Moreover, it is linked to high blood pressure, and metabolic and cardiovascular diseases [5,6]. Patients with ischemic heart disease (IHD), heart failure, arrhythmias, cerebrovascular diseases, and type II diabetes are among the high-risk groups for OSA [6,7,8]. Numerous studies have demonstrated that OSA is a risk factor for complications both before and after surgery [9,10]. According to the American Academy of Sleep Medicine (AASM), approximately 5% of women and 14% of men in the United States are affected by sleep apnea, with the majority of cases remaining undiagnosed (around 80%) [11]. The estimated annual cost associated with undiagnosed sleep apnea ranges from USD 130 billion to USD 150 billion approximately [11,12,13], but timely diagnosis of apnea can potentially save up to USD 100.1 billion [11].

Laboratory polysomnography (PSG) is the most commonly used diagnostic method for sleep apnea, involving a patient spending a night or two in a sleep laboratory with electrodes and wires attached to record physiological signals such as electrocardiogram (ECG), electroencephalogram (EEG), electromyography (EMG), electrooculogram (EOG), blood oxygen saturation (SpO_2_), airflow, and respiratory effort [14,15]. PSG requires the presence of a sleep expert to monitor and analyze the signals, making it a time-consuming and expensive technique. The complex setup and discomfort caused by sensors may result in overestimation or underestimation of the severity of sleep apnea. Therefore, there is a strong need for an alternative to laboratory PSG that is more convenient and less intrusive. In the literature, several artificial intelligence (AI)-based detection techniques have been proposed as alternatives to polysomnography for automated detection of obstructive sleep apnea.

Deep learning (DL) models have become more reliable and have found applications in various aspects of healthcare, including monitoring, prediction, diagnosis, treatment, and prognosis [16,17,18,19,20]. Advanced AI/machine learning (ML) models have shown significant success in accurately detecting and predicting sleep apnea events [21,22,23,24,25,26,27,28,29,30]. However, there is a need for improved dedicated hardware in biomedical applications as DL continues to advance in terms of performance and complexity. While AI has proven capable of matching or surpassing human experts in medical diagnosis, particularly in sleep apnea detection, implementing portable and real-time detection tools on edge devices poses challenges. Developing a computationally efficient DL network for ambulatory sleep apnea detection typically requires data centers and cloud computing, which can compromise patient data privacy. Recent publications have explored alternative approaches, such as minimal sensor models, efficient ML models, dedicated hardware, or secured cloud-based solutions, to provide higher security and optimal implementation [31,32,33,34,35,36]. Energy-efficient AI/ML edge hardware is an area of ongoing research that requires further development.

AI-embedded hardware faces several significant challenges that need to be addressed for optimal performance and widespread adoption. AI algorithms demand substantial computational power for edge inference. Embedding these models in hardware devices with limited processing capabilities poses a challenge in terms of efficiently executing complex AI tasks while maintaining low power consumption. AI computation is power hungry which makes it a challenging task to strike a balance between achieving high performance and optimizing energy consumption. Moreover, a large AI model will require significant amounts of memory and storage for storing network parameters and intermediate data [37].

In this study, we adopt depth-wise separable convolution (DSC) to detect sleep apnea from raw ECG and SpO_2_ signals. DSC has been widely used in mobile computer vision tasks, such as object recognition, where recognition tasks need to be carried out in a computationally limited platform [38,39]. But the potential of DSC has not been explored for 1-D physiological signals. The objective of this study is to build a lightweight, low-parametric model for apnea detection that can be embedded in hardware for on-chip inference in a resource-constraint environment. The contributions of this study are twofold: (1) It adopts DSC for 1-D signal and demonstrates its usability in apnea detection; (2) This study proposes a lightweight apnea detection model suitable for a resource-constraint hardware system.

## 2. Materials and Methods

### 2.1. Dataset

The ECG and SpO_2_ data used in this research were obtained from the Research Resource for Complex Physiological Signals, commonly referred to as PhysioNet [40]. PhysioNet provides a comprehensive repository of physiological data from diverse clinical domains, including sleep studies. For this study, two distinct datasets were collected from PhysioNet, and their details are outlined below.

Apnea-ECG Database [41]: The dataset hosted on PhysioNet includes a total of 70 ECG (electrocardiogram) recordings and 8 SpO_2_ (blood oxygen saturation) recordings. These recordings were collected from a group of 32 subjects, consisting of 25 males and 7 females, with an average age of 43 years. The duration of each recording varied, ranging from less than 7 h to nearly 10 h. Each recording was accompanied by annotations for apnea, which were derived by human experts using simultaneously recorded respiration and related signals. The ECG signals were sampled at a frequency of 100 Hz, while the SpO_2_ signals were sampled at 50 Hz. The annotation scheme used for the Apnea-ECG database is based on minutes, where each record is divided into non-overlapping segments of one minute. Apneic activity at the beginning of each minute after the onset of sleep is annotated.

St. Vincent’s University Hospital Database [42]: The dataset known as the St. Vincent’s University Hospital Database [14] consists of 25 complete overnight polysomnograms obtained from a group of 21 male and 4 female subjects. The average age of the participants was 50 ± 10 years, ranging from 28 to 68 years, while the mean body mass index (BMI) was 31.6 ± 40 kg/m^2^, ranging from 25.1 to 42.5 kg/m^2^. The ECG (electrocardiogram) signals in this dataset were sampled at a frequency of 128 Hz, and the SpO_2_ (blood oxygen saturation) signals were sampled at 8 Hz. The dataset follows a continuous annotation scheme, providing the onset time of sleep for each recording. Additionally, for every apneic event, the dataset includes information about the onset time and duration of the activity.

### 2.2. Segmentation

The signals were divided into segments of 12 s. Since an apneic activity was marked by its persistence for at least 10 s, the use of slightly extended 12 s segments ensured that sufficient data points were captured to reliably detect and infer apneic activity. The segmentation process varied between the datasets due to differences in their annotation schemes. For the Apnea-ECG dataset, which utilized a minute-based annotation scheme, the first 12 s of each 1-min segment were retained, while the remaining duration was discarded. Since the annotations indicated the presence of apneic activities at the beginning of each minute, analyzing the initial 12 s was sufficient to determine if the segment was apneic or not. Conversely, for the St. Vincent’s University Hospital dataset, each recording was partitioned into segments of 12 s. Based on the provided information about the onset and duration of apneic activities, any segment containing at least 10 s of apneic activity was classified as apnea. Segments with apneic activity lasting less than 10 s or no activity at all were considered normal.

The number of data points within each segment was determined by the sampling rate of the respective signal. Due to variations in sampling rates between the datasets, there was a discrepancy in the number of data points per segment. For example, an ECG segment of 12 s from the Apnea-ECG dataset contained 1200 data points, while a corresponding segment from the St. Vincent’s University Hospital dataset comprised 1536 data points. Similarly, an SpO_2_ segment consisted of either 600 or 96 data points, depending on the dataset. To ensure consistency in input shape for the classifier, the ECG signal from the St. Vincent’s University Hospital dataset was downsampled. This downsampling process aimed to match the segment length of 1200 data points, aligning it with the segment length in the Apnea-ECG dataset. Likewise, the SpO_2_ signal in the Apnea-ECG dataset was downsampled to achieve a segment length of 96 data points.

### 2.3. Data Augmentation and Class Balancing

Due to the class imbalance in the dataset, with the majority of signal segments belonging to the normal class, a technique called synthetic minority oversampling (SMOTE) was employed to address this issue [43]. SMOTE involves selecting a random instance from the minority class, denoted as ‘a’, and identifying its k nearest neighbors within the minority class. From these neighbors, one instance, denoted as ‘b’, is chosen, and synthetic instances are created by generating random points in the feature space between ‘a’ and ‘b’. The synthetic instances were formed through a convex combination of ‘a’ and ‘b’. In this study, SMOTE was applied to generate synthetic data specifically for the apnea class, with a value of k set to 5.

Data augmentation is a commonly employed technique in machine learning that involves applying diverse transformations to the original training data to generate additional training samples without the need to collect new data [44]. By generating new samples, data augmentation enhances the generalization capability and robustness of a machine learning model, as it exposes the model to different variations in the input. In this particular study, the data augmentation approach involved flipping the signal segments. Since an apneic event is characterized by changes in blood-oxygen levels and heart rate, it was assumed that flipping the segments would preserve the spatial information while introducing variations into the original dataset. This augmentation process aimed to facilitate better generalization of the machine learning model. The original set of segments was divided into training, validation, and testing sets, with a ratio of 8:1:1. The class balancing and augmentation techniques were applied to the training set only. The detailed distribution can be found in the Results section.

### 2.4. Depth-Wise Separable Convolution (DSC)

**Figure 1:**
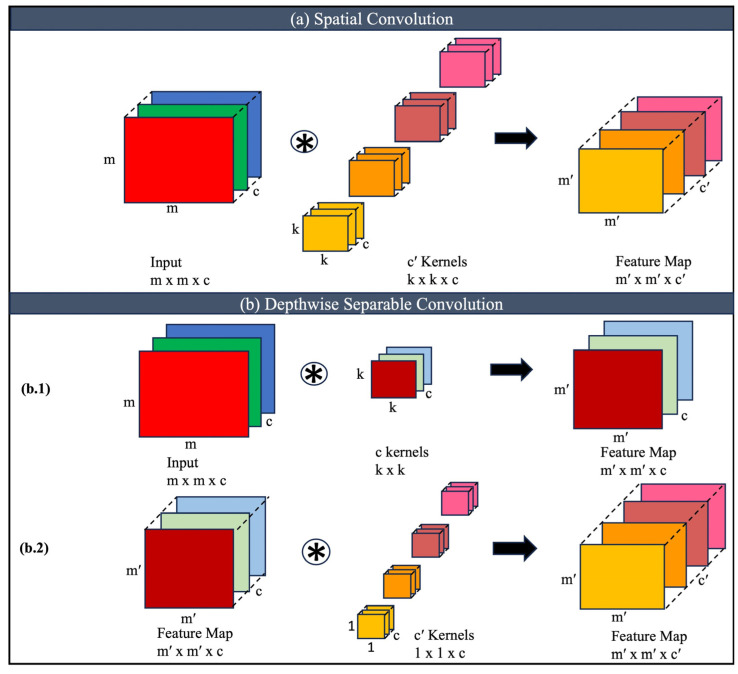
Spatial convolution and depth-wise separable convolution illustrated for multichannel 2D inputs. (**a**) Spatial Convolution, (**b**) (**b.1**) Depthwise Convolution, (**b.2**) Pointwise Convolution.

Spatial convolution (SC) and depth-wise separable convolution (DSC) are two techniques commonly used in convolutional neural networks (CNNs) for image processing tasks [38,39,45]. Although both approaches aim to reduce computational costs, they differ in their underlying operations and characteristics. Figure 1 illustrates the application of SC and DSC operation on a multichannel 2D input. SC is the conventional form of convolution used in CNNs. It involves convolving an input image with a set of learnable filters or kernels. Each filter slides across the input image, computing the element-wise dot product between its weights and the corresponding patch of the image. This process generates a feature map that represents the responses of the filters to different patterns in the input. SC performs a full-depth convolution, where each input channel is convolved with each filter independently as shown in Figure 1a. The number of parameters in spatial convolutions depends on the size of the filters and the number of input and output channels. However, spatial convolutions are computationally expensive, particularly when the input has a large number of channels, as the computation is repeated for each channel.

Depth-wise separable convolution is a variant of convolution that decomposes the process into two stages: depth-wise convolution and pointwise convolution (Figure 1b). In the depth-wise convolution stage, each input channel is convolved independently with its corresponding filter. This operation is similar to applying a separate spatial filter to each input channel, generating a set of intermediate feature maps. Depth-wise convolution reduces the computational cost compared to spatial convolution by performing convolutions on each input channel separately as illustrated in Figure 1(b.1). The number of parameters in the depth-wise convolution stage depends on the size of the filters and the number of input channels but is significantly lower than in spatial convolution.

As shown in Figure 1(b.2), after the depth-wise convolution, a pointwise convolution is performed. It applies a 1 × 1 filter to the intermediate feature maps, combining the information from different channels. This step aims to capture cross-channel interactions and generate the final output feature maps. Pointwise convolution operates on the concatenated output of the depth-wise convolution stage and uses 1 × 1 filters to combine information from different channels, creating complex interactions between channels. The number of pointwise filters determines the number of output channels in the final feature maps. Depth-wise separable convolution offers several benefits over spatial convolution. It reduces computational costs, requires less memory, and can achieve comparable or even improved performance.

According to Figure 1a, for an input of size *m × m × c* and a kernel size of *k × k × c*, total number of weights for *c*’ such kernels is as follows:

(1) $$W_{SC}=k\times k\times c\times cc^{\prime }$$

And the total number of operations is as follows:

(2) $$O_{SC}=m\times m\times k\times k\times c\times cc^{\prime }$$

For computational simplicity, the height and width of the output feature map were considered to be the same as the input (*m = m′*) and the stride was considered to be 1. Similarly, the total number of weights and operations for DSC in Figure 1b can be expressed as the following:

(3) $$W_{DSC}=k\times k\times c+c\times cc^{\prime }$$

(4) $$O_{DSC}=m\times m\times k\times k\times c+m\times m\times c\times cc^{\prime }$$

Thus, the total number of weights and operations are reduced by DSC and the reduction factor can be calculated as the following:

(5) $$R_{W}=\frac{W_{DSC}}{W_{SC}}=\frac{1}{c}+\frac{1}{K^{2}}$$

(6) $$R_{O}=\frac{O_{DSC}}{O_{SC}}=\frac{1}{c}+\frac{1}{K^{2}}$$

### 2.5. Proposed Network Architecture

**Figure 2:**
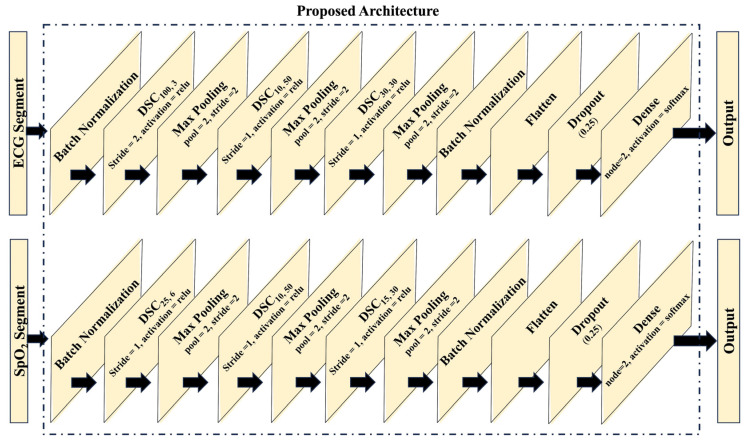
Spatial convolution and depth-wise separable convolution illustrated for multichannel 2D inputs.

Each SC layer of the base classifier was replaced by a DSC layer. Figure 2 illustrates the proposed architecture of the individual models. Each DSC layer is represented by DSC*_k,c′_* notation where *k* represents the kernel size and *c* represents the number of channels in the output feature maps as shown in Figure 1. In the case of the ECG-based model, the input is initially subjected to batch normalization, followed by the application of three DSC layers. These layers possess varying configurations: the first DSC layer comprises 3 kernels with a size of 100, employing a stride of 2; the second DSC layer incorporates 50 kernels with a size of 10, and the third DSC layer encompasses 30 kernels with a size of 30. Analogously, the SpO_2_ model adopts a parallel architecture, employing three DSC layers. Specifically, the first DSC layer consists of 6 kernels with a size of 25, the second layer integrates 50 kernels with a size of 10, and the third layer encompasses 30 kernels with a size of 15. Subsequent, to each DSC layer, a maxpooling layer with a size and stride of 2 is applied. Following the final maxpooling layers, flatten layers are employed, accompanied by dropout layers with a ratio of 0.25. The output layer of both the ECG and SpO_2_ models assumes a dense configuration, comprising two neurons with softmax activation. It is important to note that all other layers within the architecture adopt the rectified linear unit (ReLU) activation function.

**Figure 3:**
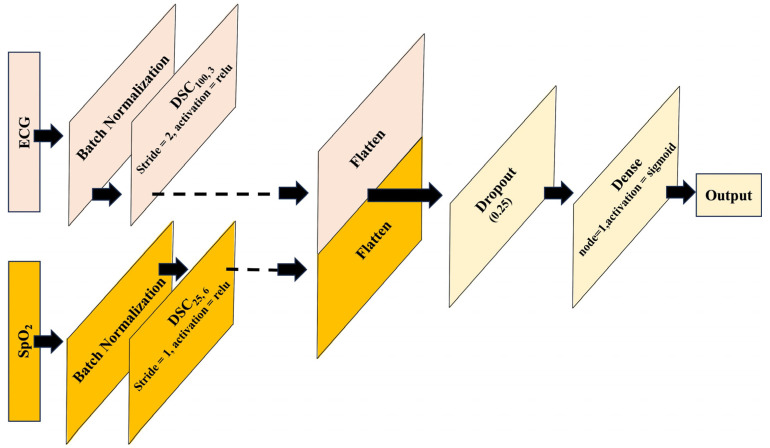
Fusion of models using transfer learning approach.

Transfer learning is a machine learning technique that leverages knowledge gained from training one model on a specific task and applies it to a different but related task [46,47]. It involves reusing the learned features or representations from a pre-trained model and using them as a starting point for training a new model on a different task or dataset. The idea behind transfer learning is that the knowledge acquired by a model during training on a large and diverse dataset can be useful for solving related problems, even if the new task or dataset is different from the original one. Instead of training a model created from scratch, which can be computationally expensive and requires a large amount of labeled data, the transfer of learning allows us to benefit from the knowledge already captured by pre-trained models. In this study, the pre-trained ECG and SpO_2_-based models were taken and concatenated at the flatten layer that creates the proposed fusion model which is illustrated in Figure 3.

## 3. Results

The ECG and SpO_2_ recordings were acquired from Apnea-ECG and St. Vincent’s University Hospital Database [41,42]. Since an apneic activity is marked by its persistence for at least 10 s, the signals were divided into segments of 12 s to ensure that each segment has sufficient data points for accurate inference. The distribution of the 12 s segments is presented in Table 1. The segmentation of the signals revealed a class imbalance, with approximately 80% of the segments belonging to the normal class for the ECG signal. Similarly, around 91% of the segments from the SpO_2_ signal were classified as normal. To address this significant imbalance, a technique called the synthetic minority oversampling technique (SMOTE) was employed, followed by an augmentation method that doubled the total number of segments [43]. In this augmentation technique, each segment was flipped to increase the number of training data for better generalization of the model. The table provides details on the distribution of signal segments in the training, validation, and test sets (8:1:1). Upon closer examination, it is evident that the number of segments obtained from the SpO_2_ signal was lower than that of the ECG signal. This disparity can be attributed to the Apnea-ECG dataset containing only 8 SpO_2_ recordings compared to the 70 ECG recordings, resulting in a smaller number of SpO_2_ segments.

**Figure 4:**
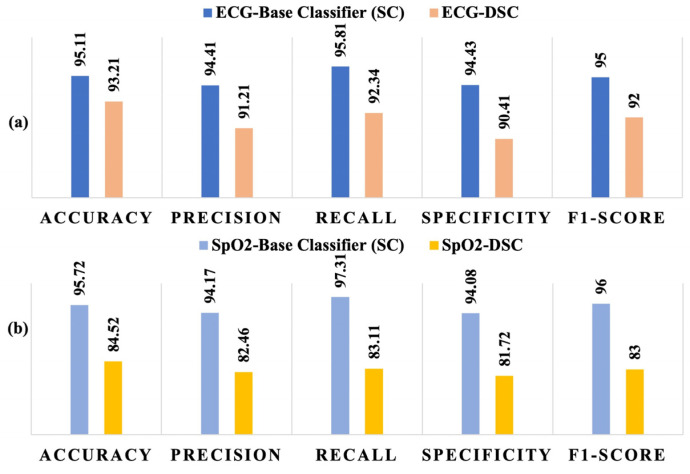
Performance comparison of the baseline classifier and the proposed DSC-based classifier for: (**a**) ECG signal and (**b**) SpO_2_ signal.

John et al. proposed a CNN model for OSA detection using raw physiological signals from multiple sensors, which has been adopted as the baseline classifier in this study [48]. In this study, we replaced each spatial convolution (SC) layer of the baseline classifier with a DSC layer to explore the potential of DSC in reducing computational complexity while maintaining performance. Figure 4 illustrates the performance metrics obtained by the baseline SC model and the proposed DSC implementation for both ECG and SpO_2_ signals. It is evident from the results that the baseline model outperformed the DSC implementation for both types of signals across all reported performance metrics, including accuracy, precision, recall, F1 score, and specificity. However, the performance of the DSC model for ECG signals remained competitive, as shown in Figure 4a, with performance metrics exceeding 90% for both networks. This demonstrates that while the DSC implementation sacrifices some accuracy compared to the baseline, it still provides reliable results for ECG signal-based OSA detection. Conversely, the DSC model showed a more significant decline in performance for SpO_2_ signals, as presented in Figure 4b. The performance metrics, including accuracy and recall, were lower than those of the baseline model, indicating that the DSC-based architecture is less effective for SpO_2_ signal processing in this context. The results suggest that further optimization might be necessary to enhance its suitability for specific physiological signals like SpO_2_.

**Figure 5:**
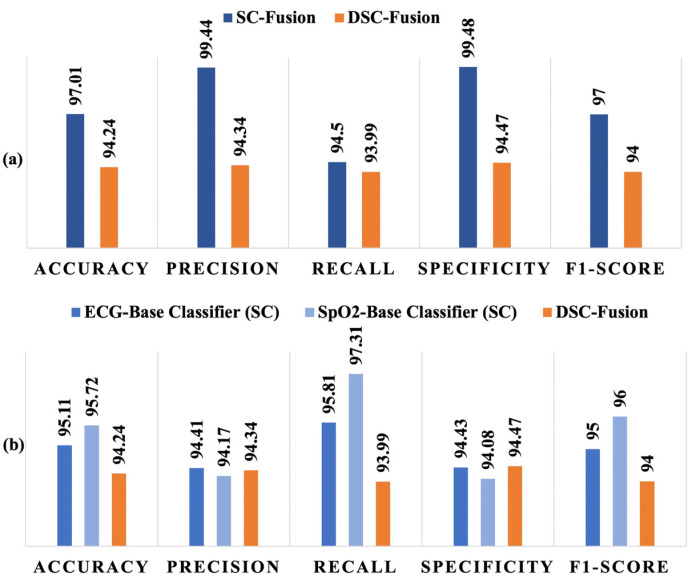
Performance comparison of the DSC-based fusion model with (**a**) SC-based fusion model and (**b**) base classifiers using individual signals.

Furthermore, two fusion models were developed to explore the possibility of more accurate inference. One of the models was the fusion of the baseline classifiers: SC-based ECG model and SC-based SpO_2_ model. The second one was the fusion of the DSC-based models. Figure 5a shows the performance comparison of SC-fusion and DSC-fusion models and Figure 5b compares the performance of the DSC-fusion with the individual baseline classifiers. Although both models had ~94% recall value, the SC-fusion model outperformed the DSC-fusion model in all other performance metrics. However, a significant improvement in the performance was observed when DSC-fusion was compared with the individual baseline classifiers. Accuracy, precision, and specificity yielded by the DSC-fusion and individual baseline models were ~95%, ~94%, and ~94%, respectively. Although the recall (~94%) value and F_1_-score (94) of the DSC-fusion model were lower than those of the baseline models, they were clearly higher than the pre-fusion DSC-based classifier shown in Figure 4.

There are reports of evaluating the computational complexities of models by quantifying the number of multiplications and additions needed per second [48,49]. This calculation relied on a straightforward filtering calculation count for the convolution layers [50]. As for the maxpooling layers, the process of selecting the maximum value was approximated as an addition operation. The estimation of the overall energy consumption during prediction is conducted based on certain assumptions. Specifically, it is assumed that a 16-bit multiplication accumulation (MAC) operation consumes approximately 0.39 pJ of energy, as indicated by previous studies [51,52]. Additionally, a 16-bit adder is estimated to consume approximately 20 fJ of energy in 28 nm FD-SOI technology [53]. The total number of parameters and floating point operations (multiplication and addition) involved with both the base models and proposed models are shown in Table 2. Overall, the table showcases the variations in parameter counts, floating point operations, and energy consumption among different models. The use of DSC models reduces the number of parameters and floating-point operations, consequently resulting in lower memory requirements and energy consumption compared to the corresponding baseline models. Although the DSC-fusion model required the most number of parameters, floating point operations, and consequently the highest energy consumption per inference (0.27 mJ) among all the DSC-based models listed in Table 2, it required lower energy than all the SC-based baseline models. In fact, the DSC-fusion model was 9.4, 1.85, and 11.3 times more energy efficient than SC-based ECG, SpO_2_, and fusion models, respectively.

## 4. Discussion

In our previous research endeavors, our primary focus has been on optimizing hardware design to achieve energy-efficient AI inference on dedicated hardware platforms. We have dedicated our efforts to developing innovative architectures and algorithms that minimize energy consumption during AI inference tasks. Our work has involved, exploring various techniques such as hardware acceleration, custom circuit design, and low-power optimizations, all aimed at enhancing the energy efficiency of AI hardware systems [54,55]. In our current work, we have shifted our attention towards optimizing the network itself with the goal of further improving energy efficiency. Specifically, we are actively working on reducing the number of parameters and floating-point operations (FLOPs) within the network architecture. This reduction in parameters and FLOPs not only enhances the computational efficiency of the network but also facilitates better hardware design, as it enables the development of specialized hardware architectures tailored to the specific requirements of the optimized network.

DSC reduces the computational complexity associated with conventional convolutional operations. However, further simplification can be achieved by applying additional techniques such as pruning, quantization, and other optimization methods. Pruning involves identifying and removing redundant or insignificant connections within the DSC architecture. This can be accomplished by setting small weights or pruning entire channels that contribute minimally to the network’s overall performance. Pruning not only reduces the model’s memory footprint but also decreases the number of computations required during inference, leading to improved efficiency. Quantization is another technique that can be applied to simplify DSC models. It involves reducing the precision of weights and activations from floating-point to lower-bit representations, such as fixed-point or binary values. By quantizing the parameters, the memory requirements and computational complexity of the DSC network can be significantly reduced. Additionally, specialized hardware accelerators can be leveraged to exploit the efficiency of quantized operations. These simplification methods strike a balance between model size, computational requirements, and performance, enabling the deployment of lightweight and energy-efficient DSC models without sacrificing accuracy.

This study has several limitations. First, the signals used in this study, ECG and SpO_2_, may not fully capture all relevant physiological markers for sleep apnea detection, such as airflow or respiratory effort, potentially limiting the model’s diagnostic capability in more complex cases. Additionally, the dataset lacks diversity in terms of population, which could affect the generalizability of the models when applied to broader, more heterogeneous populations with varying degrees of apnea severity and comorbid conditions. Moreover, we did not perform a statistical analysis to compare the models because our conclusion is not centered on demonstrating that the DSC model is equivalent to the SC model in terms of performance. Rather, the conclusion highlights that, despite a slight reduction in performance, the DSC-based model’s energy efficiency outweighs this drawback, making it a preferable choice for scenarios where power consumption is a primary concern. For future work, incorporating more diverse datasets and additional physiological signals could improve the model’s accuracy and robustness. Finally, implementing these models in real-time monitoring devices, such as wearables, with personalized adaptive algorithms could enhance their practical utility in detecting apnea in diverse and real-world settings.

**Figure 6:**
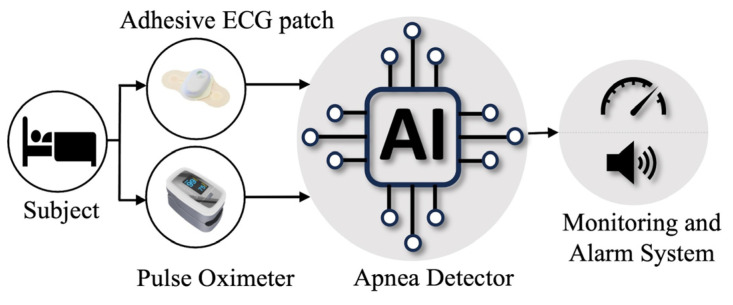
Schematic Diagram of the proposed AI-based apnea detection system in healthcare settings.

AI-driven apnea detection systems have the potential to transform healthcare by seamlessly integrating into various settings. In sleep clinics, these systems automate sleep study analysis, saving time and improving diagnostic accuracy. Instead of manually reviewing hours of recorded sleep data, AI algorithms can quickly and accurately identify apnea events, allowing clinicians to focus on interpreting the results and designing appropriate treatment plans. In hospitals, AI algorithms continuously monitor at-risk patients, promptly detecting apnea episodes and enabling timely intervention. The real-time monitoring can enhance patient safety and facilitate early intervention, reducing the risk of complications associated with apnea, especially with patients recovering from surgery or in critical care units. Figure 6 illustrates the AI-driven apnea detection system using ECG and oxygen saturation signals in healthcare applications. Moreover, wearable devices equipped with AI can track sleep patterns and detect apnea events in home-based care, allowing for remote monitoring and personalized interventions. Overall, AI-driven apnea detection systems enhance diagnostic efficiency, patient safety, and accessibility, revolutionizing the management of apnea in healthcare.

## 5. Conclusions

Although accurate and precise detection of a clinical event is the primary objective of a diagnosis or monitoring tool, achieving higher performance is often the most challenging task for an AI-embedded system due to resource constraints. This study proposes an energy-efficient and low-parametric model using DSC that requires ~2–~11 times lower storage capacity and computations per inference. DSC is widely used in mobile computer vision tasks; however, its potential in clinical application on 1-D signal was unexplored. The adoption of DSC in AI-embedded system for apnea detection can strike a balance between performance and computational requirements. Although the SC-based fusion model outperformed the DSC implementation, the DSC-based model is still preferable due to its high energy efficiency with acceptable performance.

## Figures and Tables

**Table 1:** Distribution of signal segments with a processing window of 12 s.

	ECG			SpO_2_		
	Train	Validation	Test	Train	Validation	Test
Total	214,264	8267	8264	152,364	5216	5222
Apnea	107,132	1572	1570	76,182	456	460
Normal	107,132	6695	6694	76,182	4760	4762

**Table 2:** Complexity analysis of the baseline model and the proposed model and energy requirement per inference.

Model	Parameters	Multiplication	Addition	Energy (μJ)
SC-ECG	51,389	6,534,116	6,546,647	2.55
DSC-ECG	7872	579,439	580,311	0.23
SC-SpO_2_	26,702	1,270,016	1,272,876	0.50
DSC-SpO_2_	3693	103,866	105,432	0.04
SC-Fusion	78,089	7,809,352	7,824,743	3.05
DSC-Fusion	11,563	683,303	684,721	0.27

## Data Availability

The original data used in this study are openly available in https://physionet.org/ (accessed on 5 August 2023).
